# By Improving Regional Cortical Blood Flow, Attenuating Mitochondrial Dysfunction and Sequential Apoptosis Galangin Acts as a Potential Neuroprotective Agent after Acute Ischemic Stroke 

**DOI:** 10.3390/molecules171113403

**Published:** 2012-11-09

**Authors:** Shaojing Li, Chuanhong Wu, Li Zhu, Jian Gao, Jing Fang, Defeng Li, Meihong Fu, Rixin Liang, Lan Wang, Ming Cheng, Hongjun Yang

**Affiliations:** 1 Institute of Chinese Materia Medica, China Academy of Chinese Medical Sciences, Beijing 100700, China; 2 Jiangxi University of Traditional Chinese Medicine, Nanchang, Jiangxi 330006, China; 3 College of Pharmaceutical Science, Hebei University, Baoding, Hebei 071002, China

**Keywords:** galangin, mitochondria, cerebral ischemia

## Abstract

Ischemic stroke is a devastating disease with a complex pathophysiology. Galangin is a natural flavonoid isolated from the rhizome of *Alpina officinarum* Hance, which has been widely used as an antioxidant agent. However, its effects against ischemic stroke have not been reported and its related neuroprotective mechanism has not really been explored. In this study, neurological behavior, cerebral infarct volumes and the improvement of the regional cortical blood flow (rCBF) were used to evaluate the therapeutic effect of galangin in rats impaired by middle cerebral artery occlusion (MCAO)-induced focal cerebral ischemia. Furthermore, the determination of mitochondrial function and Western blot of apoptosis-related proteins were performed to interpret the neuroprotective mechanism of galangin. The results showed that galangin alleviated the neurologic impairments, reduced cerebral infarct at 24 h after MCAO and exerted a protective effect on the mitochondria with decreased production of mitochondrial reactive oxygen species (ROS). These effects were consistent with improvements in the membrane potential level (Δψm), membrane fluidity, and degree of mitochondrial swelling in a dose-dependent manner. Moreover, galangin significantly improved the reduced rCBF after MCAO. Western blot analysis revealed that galangin also inhibited apoptosis in a dose-dependent manner concomitant with the up-regulation of Bcl-2 expression, down-regulation of Bax expression and the Bax/Bcl-2 ratio, a reduction in cytochrome c release from the mitochondria to the cytosol, the reduced expression of activated caspase-3 and the cleavage of poly(ADP-ribose) polymerase (PARP). All these data in this study demonstrated that galangin might have therapeutic potential for ischemic stroke and play its protective role through the improvement in rCBF, mitochondrial protection and inhibiting caspase-dependent mitochondrial cell death pathway for the first time.

## 1. Introduction

Ischemic stroke is one of the most frequent causes of death and neurological disability [1]. Cerebral ischemia is a common pathological state and has been revealed as one of the causes or key factors contributing to stroke [2]. Moreover, reduced cerebral blood flow has recently been suggested as a predictive marker for the progression of stroke [3]. Therefore, restoring the blood supply to the brain as quickly as possible has been considered the most important strategy to treat ischemic stroke.

Accumulating evidence has shown that mitochondrial dysfunction plays a pivotal role in cerebral ischemia disorders through its effects on the brain, including energy failure, intracellular calcium overload, oxidative stress and apoptosis. It is essential to evaluate the mitochondrial functions in physiological and pathological conditions to provide more information for the development of new drugs. Moreover, scattered regional cerebral blood flow (rCBF) decreases and increases have been reported in mitochondrial disorders [4]. Currently, assays for membrane integrity, membrane fluidity, mitochondrial membrane potential [5] are commonly used for determining mitochondria function. 

The dietary flavonoid galangin is one of a group of naturally occurring compounds in plants and is present in high concentrations in the rhizome of *Alpinia officinarum* Hance, which has been used in China for centuries as both a spice and a traditional Chinese medicine for various ailments [6]. Galangin has multiple bioactivities and affects many cell systems. In addition to its anti-oxidant, anti-mutagenic, and anti-tumor effects, galangin has also been shown to possess anti-inflammatory, anti-microbial, and anti-viral activities in a variety of *in vitro* and *in vivo* systems [7,8,9]. Furthermore, galangin has vasodilation [10], anti-ischemic, and anti-oxidant properties, which may reduce the risk of coronary heart disease and improve endothelial cell function [11]. In a recent study, investigators found that galangin inhibits acetylcholinesterase activity *in vitro* and might be of potential use for the treatment of Alzheimer’s disease [12]. Another report showed that galangin was a modulator of vascular smooth muscle Ca(v)1.2 channels, which might be valuable in the treatment of hypertension and stroke [13]. But there is no experimental evidence for the protective effects of galangin on stroke.

Previous reports have identified galangin as an anti-oxidant agent that is possibly associated with the functional regulation of mitochondria. However, the mitochondrial protection and anti-apoptotic mechanisms of galangin have also not been reported for cerebral ischemia. In this study, a widely accepted model of focal cerebral ischemia induced by middle cerebral artery occlusion (MCAO) in rats was used to evaluate the possible protective effects of galangin on ischemic brain injury [14]. Furthermore, the related mechanisms underlying its effects, including those associated with reduced rCBF, mitochondrial dysfunction, oxidative stress and apoptosis, were also investigated.

## 2. Results and Discussion

### 2.1. Neurological Defects

**Figure 1:**
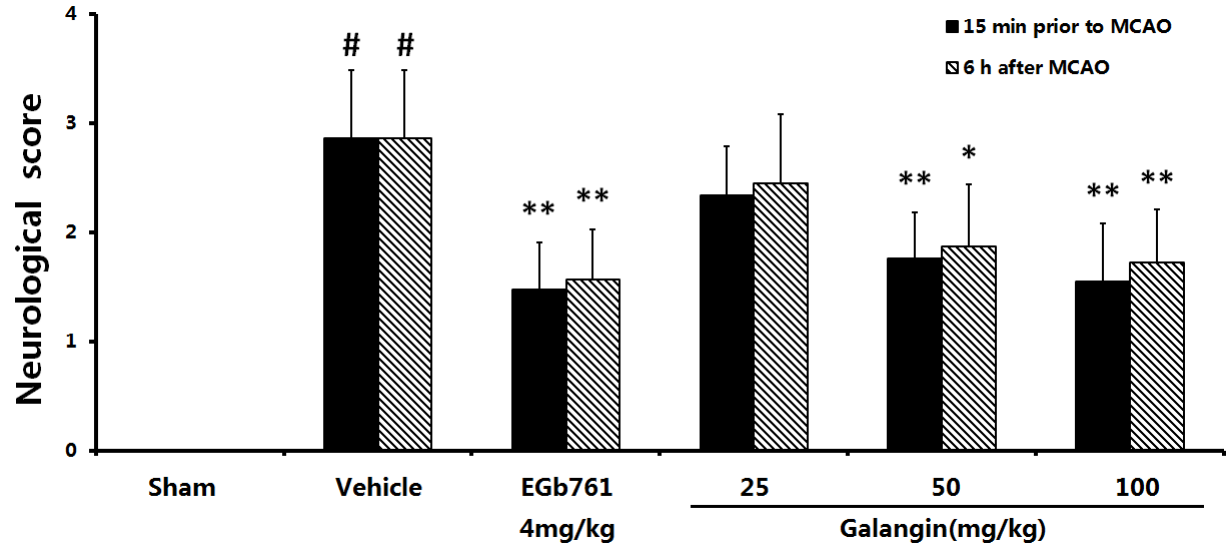
Effects of galangin on neurological deficits induced by MCAO. EGb761 and galangin were both administered by i.g. 15 min prior to MCAO and 6 h after MCAO. The values were expressed as the means ± SD (n = 10) and the data were analyzed by one-way ANOVA. ^##^ *p* < 0.01, ^#^ *p* < 0.05 *versus* the sham group; ** *p* < 0.01, * *p* < 0.05 *versus* the vehicle control.

Middle cerebral artery occlusion was performed on the left side, and 24 h later, right hind paresis was observed in rats compared to the contralateral side, as shown in Figure 1. At PM and AM, the mean neurological score in the vehicle-treated group (2.87 ± 0.62) were significantly (*p* < 0.05) higher than the sham groups, indicating a neurological defect after the MCAO. In the EGb761 treated groups and the galangin- (50 mg·kg^−1^, 100 mg·kg^−1^) treated groups, the neurological deficits significantly improved (*p* < 0.05 or *p* < 0.01) compared to the vehicle-treated group. However, in the galangin-treated (25 mg·kg^−1^) group, no improvement of neurological defect was observed compared to the vehicle-treated MCAO group. 

### 2.2. Cerebral Infarct Size

**Figure 2:**
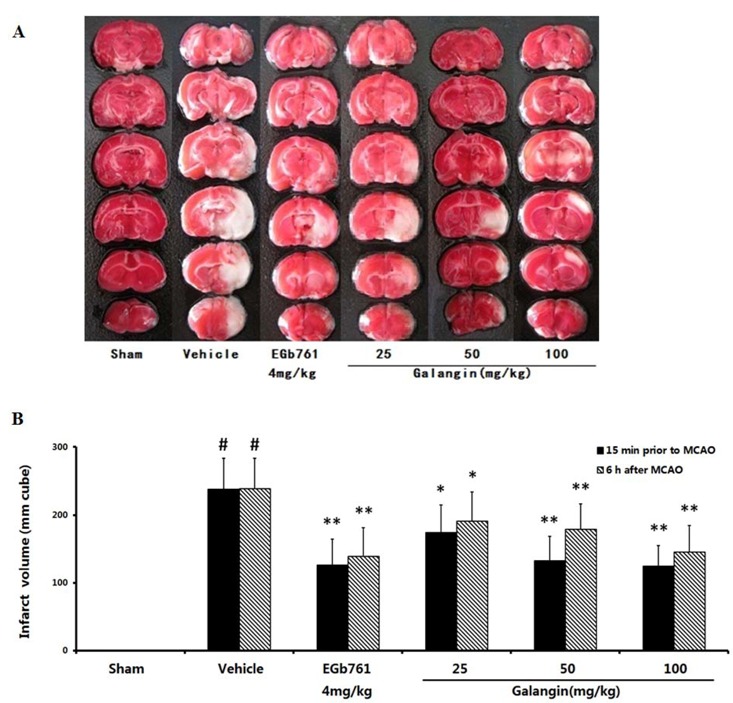
Effects of galangin on the infarct volume of rats induced by MCAO. (**A**) Illustrative coronal sections showing the infarct volumes in the cerebral hemisphere as adistinct, pale stained area in the rats subjected to ischemia and the attenuation of the infarct volume by treatment with galangin; (**B**) The effects of galangin on the infarct volume of rats induced by MCAO. EGb761 and galangin were both administered by i.g. 15 min prior to MCAO and 6 h after MCAO. The values were expressed as the mean ± SD (n = 10), and the data were analyzed by one-way ANOVA. ^##^ *p* < 0.01, ^#^ *p* < 0.05 *versus* the sham group; ** *p* < 0.01, * *p* < 0.05 *versus* the vehicle control.

The ischemia produced a marked infarct as a result of the MCAO in the serial coronal brain sections. At PM and BM, the mean infarct volumes in the vehicle-treated group were 238.17 ± 45.84 mm^3^ (*p* < 0.05) (Figure 2). Oral administration of EGb761 and galangin (50 and 100 mg·kg^−1^) significantly reduced the infarct volume (*p* < 0.01) compared to the vehicle control. The group treated with galangin (25 mg·kg^−1^) also had a reduction in the infarct volume (*p* < 0.05).

### 2.3. Regional Cortical Blood Perfusion

**Figure 3:**
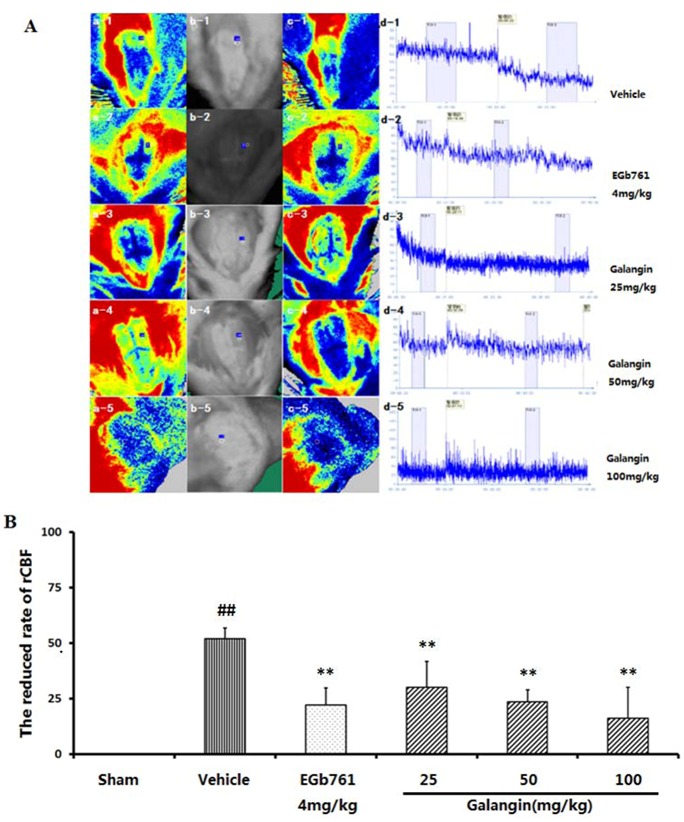
Effects of galangin on the rCBF of rats induced by MCAO. EGb761 and galangin were both administered by i.g.15 min prior to MCAO. (**A**) Detection of cerebral blood flow. The cerebral blood flow perfusion color image (Figure 3A.a1–5 and c1–5), detected position image (Figure 3 3A.b1–5), and blood flow time window (Figure 3A.d1–5) were presented (1, Vehicle control; 2, EGb761-treated group; 3–5: galangin- (25, 50 and 100 mg·kg^−1^) treated group); (**B**) The reduced rate of rCBF. The values were expressed as the mean ± SD (n = 5), and the data were analyzed by one-way ANOVA. ^##^ *p* < 0.01, ^#^ *p* < 0.05 *versus* the sham group; ** *p* < 0.01, * *p* < 0.05 *versus* the vehicle control.

We detected the cerebral blood flow with the Perfusion Speckle Imager (PERIMED, Stockholm, Sweden), and the picture is presented in Figure 3A. The reduction of cerebral blood flow following MCAO in the vehicle-treated group was 51.95 ± 4.71 compared to the sham group (*p* < 0.01) (Figure 3B). The cerebral blood flows of the EGb761- and galangin- (25, 50 and 100 mg·kg^−1^) treated groups had a significant enhancement (*p* < 0.01) compared to the vehicle-treated group after MCAO. 

### 2.4. Measurement of Mitochondrial Function

**Figure 4:**
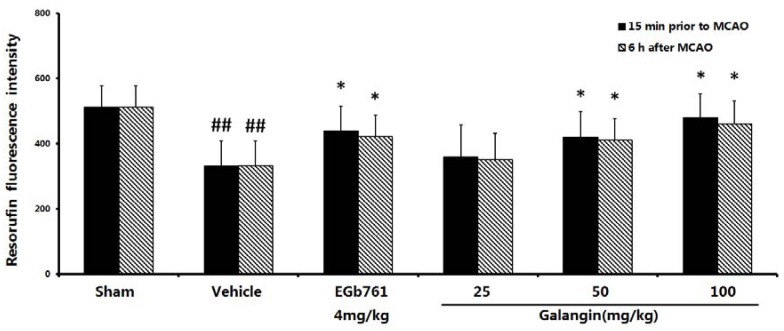
Effects of galangin on mitochondrial function caused by MCAO. EGb761 and galangin were both administered by i.g. 15 min prior to MCAO and 6 h after MCAO. The values were expressed as the mean ± SD (n = 10), and the data were analyzed by one-way ANOVA. ^##^ *p* < 0.01, ^#^ *p* < 0.05 *versus* the sham group; ** *p* < 0.01, * *p* < 0.05 *versus* the vehicle control.

Mitochondrial function was evaluated by measuring the fluorescence intensity of resorufin. As shown in Figure 4, at PM, the mitochondrial viability was significantly changed in the vehicle-treated group. This value decreased from 511.83 ± 65.72 to 332.26 ± 77.73 as a result of the MCAO (*p* < 0.01). With the exception of the low-dose group of galangin, the galangin-treated groups at doses of 50 mg·kg^−1^ and 100 mg·kg^−1^ and the EGb761-treated group at a dose of 4 mg·kg^−1^ improved the mitochondrial function to a higher level (*p* < 0.05) compared to the vehicle control. At AM, the vehicle-treated group had significantly decreased mitochondrial function (*p* < 0.01) compared to the sham-treated group. The galangin-treated group at a dose of 50 mg·kg^−1^ and 100 mg·kg^−1^ and the EGb761-treated group at a dose of 4 mg·kg^−1 ^improved the mitochondrial function to a higher level (*p* < 0.05). However, there was no significant improvement in the galangin-treated group at doses of 25 mg·kg^−1^. 

### 2.5. Measurement of Mitochondrial Swelling

The decrease in the OD at 520 nm indicates mitochondrial swelling. Mitochondrial swelling was more serious in the vehicle control compared to the sham group. The group treated with EGb761 and galangin (25 mg·kg^−1^, 50 mg·kg^−1^ and 100 mg·kg^−1^) showed significantly (*p* < 0.01, PM and AM) decreased amounts of mitochondrial swelling (Table 1).

**Table 1:** Absorbency of slope absolute value.

Group	Dose (mg·kg^−1^)	n	Swelling/A_520_ (Slope absolute value)	
			15 min prior to MCAO	6 h after MCAO
Sham	-	10	0.053 ± 0.012	0.035 ± 0.067
Vehicle	-	10	0.402 ± 0.008 ^##^	0.476 ± 0.011 ^##^
EGb761	4	10	0.093 ± 0.014 **	0.064 ± 0.007 **
Galangin	25	10	0.389 ± 0.021 **	0.355 ± 0.010 **
Galangin	50	10	0.187 ± 0.010 **	0.167 ± 0.008 **
Galangin	100	10	0.093 ± 0.008 **	0.089 ± 0.013 **

The values were expressed as the mean ± SD (n = 10) and the data were analyzed by one-way ANOVA. ^##^ *p* < 0.01, ^#^ *p* < 0.05 *versus* the sham group; ** *p* < 0.01, * *p* < 0.05 *versus* the vehicle control.

### 2.6. Mitochondrial Membrane Fluidity Measurement

**Figure 5:**
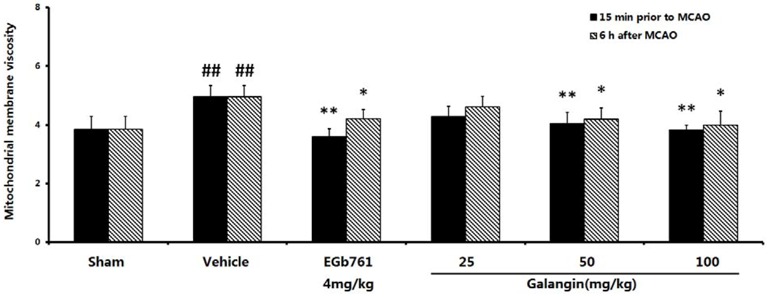
Effects of galangin on mitochondrial membrane viscosity caused by MCAO. EGb761 and galangin were both administered by i.g. 15 min prior to MCAO and 6 h after MCAO. The values were expressed as the mean ± SD (n = 10), and the values were analyzed by one-way ANOVA. ^##^ *p* < 0.01, ^#^ *p* < 0.05 *versus* the sham group; ** *p* < 0.01, * *p* < 0.05 *versus* the vehicle control.

The mitochondrial membrane fluidity was reflected by the membrane viscosity (η). A higher η value indicates the decreased fluidity of the mitochondrial membrane. As shown in Figure 5, at PM and AM, the vehicle-treated group had a higher η values of 4.95 ± 0.38 compared to the sham group (*p* < 0.01). The decreased η value in the groups treated with EGb761 and galangin (50 mg·kg^−1^ and 100 mg·kg^−1^) was also observed (PM, *p* < 0.01; AM, *p* < 0.05) compared to the vehicle-treated group. The η of the group treated with galangin (25 mg·kg^−1^) was not significantly improved compared to the vehicle-treated group.

### 2.7. The Effect of Galangin on Mitochondria Transmembrane Potential after MCAO

#### 2.7.1. Determination with Rhodamine 123

**Figure 6:**
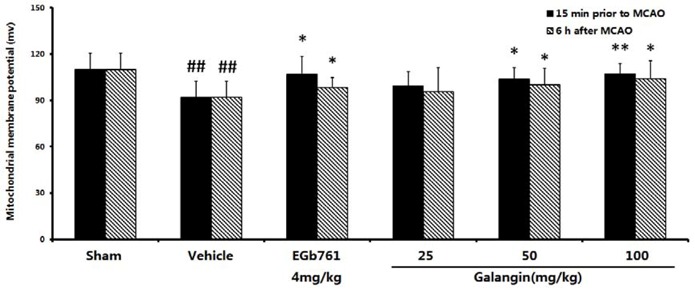
Effects of galangin on the mitochondrial membrane potential caused by MCAO. EGb761 and galangin were both administered by i.g. 15 min prior to MCAO and 6 h after MCAO. The values were expressed as the mean ± SD (n = 10), and the data were analyzed by one-way ANOVA. ^##^ *p* < 0.01, ^#^ *p* < 0.05 *versus* the sham group; ** *p* < 0.01, * *p* < 0.05 *versus* the vehicle control.

Changes in Δψ_m_ were monitored by measuring the release of rhodamine 123 which was preloaded into mitochondria. As shown in Figure 6, at PM and AM, the Δψ_m_ were significantly decreased from 109.79 ± 10.99 mv to 91.77 ± 10.56 mv as a result of the MCAO (*p* < 0.01). The EGb761-treated groups showed an enhanced membrane potential compared to the vehicle-treated group (*p* < 0.05, PM and AM). The galangin-treated groups showed an improved membrane potential (*p* < 0.01, PM, *p* < 0.05, AM) at a dose of 50 mg·kg^−1^ and 100 mg·kg^−1^. However, galangin at a dose of 25 mg·kg^−1^ (PM and AM) could not significantly improve the reduction in the mitochondrial membrane potential.

#### 2.7.2. Determination by the JC-1 Method

**Figure 7:**
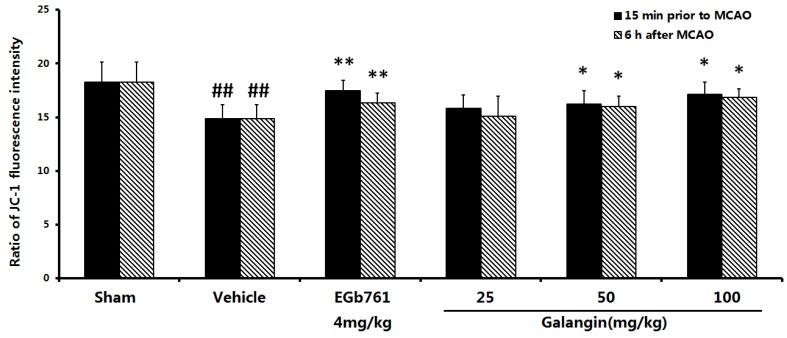
The red/green fluorescence ratio of JC-1 caused by MCAO. EGb761 and galangin were both administered by i.g. 15 min prior to MCAO and 6 h after MCAO. The values were expressed as the mean ± SD (n = 10), and the data were analyzed by one-way ANOVA. ^##^ *p* < 0.01, ^#^ *p* < 0.05 *versus* the sham group; ** *p* < 0.01, * *p* < 0.05 *versus* the vehicle control.

To measure the depolarization of the mitochondrial membrane, the mitochondrial membrane potential was measured with the probe, JC-1. The red/green fluorescence ratio of JC-1 was shown in Figure 7. At PM and AM, the vehicle-treated group had a significant decrease in this ratio, from 18.25 ± 1.88 to 14.84 ± 1.30 (*p* < 0.01) compared to the sham group. The EGb761-treated group showed an increase in the ratio to 17.46 ± 0.95 (*p* < 0.01, PM) and 16.33 ± 0.89 (*p* < 0.01, AM) compared to the vehicle-treated group. At a dose of 50 mg·kg^−1^ and 100 mg·kg^−1^, galangin increased this ratio significantly (*p* < 0.05, at PM and AM); however, galangin at 25 mg·kg^−1^ did not significantly increase the ratio.

### 2.8. Effects of Galangin on Mitochondrial ROS Levels

**Figure 8:**
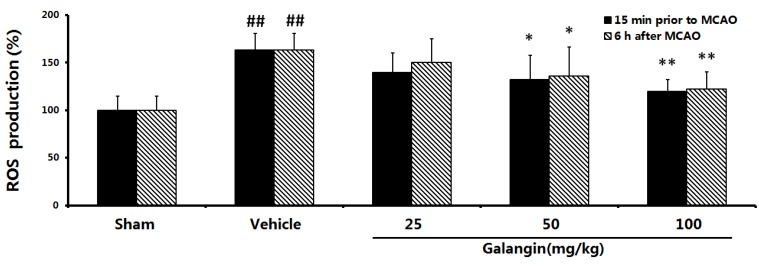
Effects of galangin on ROS generation in the brain mitochondria of MCAO rats. Galangin was administered by i.g. 15 min prior to MCAO and 6 h after MCAO. The mean mitochondrial ROS production in the sham group was set to 100%. The values were expressed as the mean ± SD (n = 10), and the data were analyzed by one-way ANOVA. ^##^ *p* < 0.01, ^#^ *p* < 0.05 *versus* the sham group; ** *p* < 0.01, * *p* < 0.05 *versus* the vehicle control.

Mitochondria are postulated to be the major source of ROS production in cells. The results of ROS generation were shown in Figure 8. Cerebral ischemia induces an increase in the production of ROS. At PM and AM, the DCF fluorescence in the MCAO rat brain mitochondria was significantly higher than the sham group (*p* < 0.01). Treatment with galangin (50 and 100 mg·kg^−1^) significantly decreased the DCF fluorescence (*p <* 0.05 or *p* < 0.01). This showed that galangin can markedly reduce ROS production in a dose-dependent manner.

### 2.9. Evaluation of the Anti-Apoptotic Effect

#### 2.9.1. Modulation of Bcl-2 and Bax Expression

**Figure 9:**
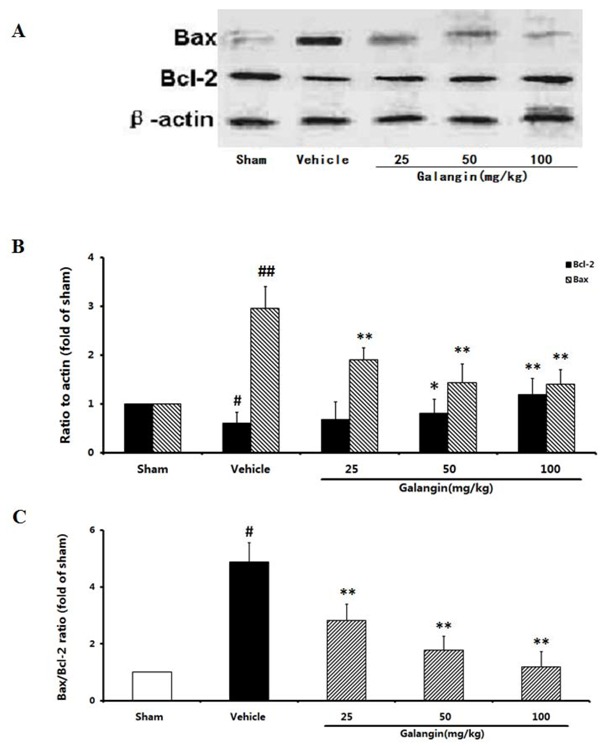
Effects of galangin on Bcl-2 and Bax expression in rat brain 24 h after MCAO. Galangin was administered by i.g. 15 min prior to MCAO. (**A**) Representative Western blots of Bcl-2, Bax and β-actin; (**B**) The quantified densitometric analysis of Bcl-2 and Bax levels; (**C**) The ratio of Bax/Bcl-2 proteins. The values were expressed as the mean ± SD for four independent experiments, and the data were analyzed by one-way ANOVA. ^##^ *p* < 0.01, ^#^ *p* < 0.05 *versus* the sham group; ** *p* < 0.01, * *p* < 0.05* versus* the vehicle control.

As shown in Figure 9, Bcl-2, a key protein that contributes to cell survival, was present at a relatively high level in the sham group and decreased in the vehicle control at 24 h after the MCAO. In contrast, the level of Bax, an important pro-apoptotic protein, increased markedly in the vehicle control. As shown in Figure 9, the ratio of Bax/Bcl-2 in the vehicle control increased significantly (4.89-fold of sham), and these may be involved in the apoptotic cell death caused by MCAO. At doses of 25–100 mg·kg^−1^, galangin reduced the up-regulation of Bax and increased the level of Bcl-2 when administrated 6 h after the MCAO. Therefore, treatment with galangin was effective in maintaining the balance between Bcl-2 and Bax.

#### 2.9.2. Modulation of Activated Caspase-3 and PARP Expression

**Figure 10:**
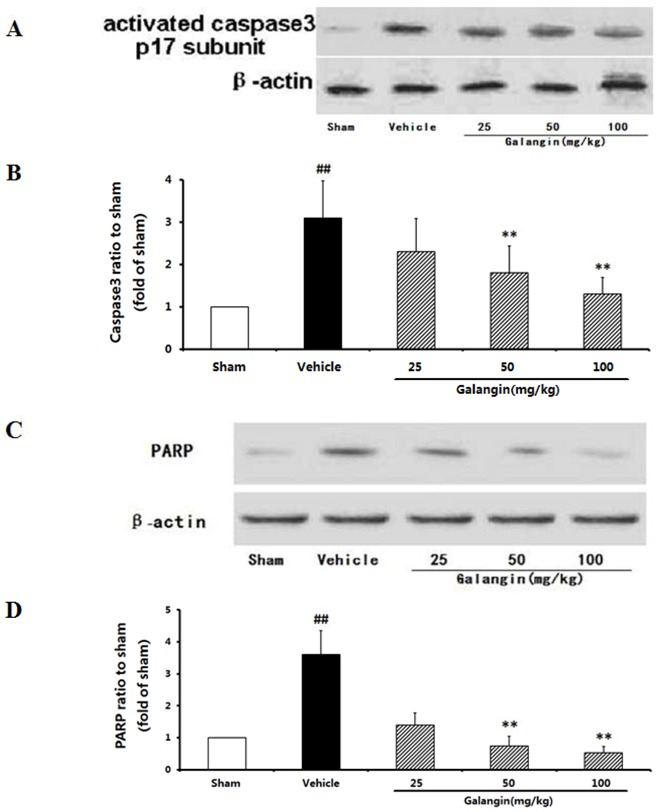
Effects of galangin on caspase-3 and PARP expression in rat brain 24 h after MCAO. Galangin was administered by i.g. 15 min prior to MCAO. (**A**) Representative western blots of caspase-3 andβ-actin; (**B**) The quantified densitometric analysis of caspase-3; (**C**) Representative western blots of PARP cleavage and β-actin; (**D**) The quantified densitometric analysis of PARP cleavage. The values were expressed as the mean ± SD for four independent experiments, and the data were analyzed by one-way ANOVA. ^##^ *p* < 0.01, ^#^ *p* < 0.05 *versus* the sham group; ** *p* < 0.01, * *p* < 0.05 *versus* the vehicle control.

Caspase-3 is an important executioner in apoptosis because it hydrolyzes a number of structural and signaling proteins involved in apoptosis, including the DNA repair enzyme, PARP. As illustrated in Figure 10B, Western blot analysis showed that the level of activated caspase-3 increased significantly after MCAO. As a result, the cleavage of PARP, an intrinsic substrate of caspase-3, also increased significantly. However, the increased activation of caspase-3 and cleavage of PARP were reduced in the galangin- (50 and 100 mg·kg^−1^) treated groups when administrated 6 h after the MCAO. The action of galangin with respect to these molecular events was most likely paralleled with its effects on apoptosis [15]. 

#### 2.9.3. Modulation of Cytochome c Expression

**Figure 11:**
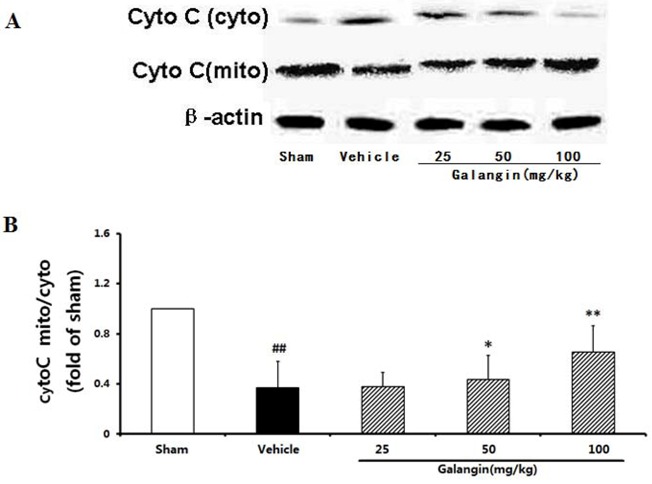
Effects of galangin on cytochrome c release from rat brain mitochondria 24 h after MCAO. Galangin was administered by i.g.15 min prior to MCAO. (**A**) Cytochrome c protein levels in the cytosolic and mitochondrial fractions; (**B**) The quantified densitometric analysis of cytochrome c. The values were expressed as the mean ± SD for four independent experiments, and the data were analyzed by one-way ANOVA. ^##^ *p* < 0.01, ^#^ *p* < 0.05 *versus* the sham group; ** *p* < 0.01, * *p* < 0.05 *versus* the vehicle control.

One of the mechanisms by which Bcl-2 blocks apoptosis is to decrease cytochrome c release from the mitochondria. As shown in Figure 11A, the significant translocation of cytochrome c had been detected in the vehicle control, in which the ratio of cytochrome c content in the mitochondrial and cytosolic fractions was approximately 0.37-fold of the sham group (*p* < 0.01) at 24 h after MCAO. Galangin treatment markedly increased the ratio (*p* < 0.01) (Figure 11B) when administrated 6 h after the MCAO. These results suggested that galangin could significantly inhibit the release of cytochrome c.

### 2.10. Discussion

It was firstly proved that galangin had positive effect on acute ischemic stroke in this study. Cerebral ischemia can cause brain injury, leading to neurology defects, cerebral infarct and neuronal death by apoptosis and/or necrosis [16]. Our results indicated that galangin at doses of 50 and 100 mg·kg^−1^ exhibited significant neuroprotective activity at 24 h after focal cerebral ischemia in a MCAO rat model, both at PM and AM. Here we demonstrated that galangin reduces infarct volumes in a rat model of focal cerebral ischemia injury. This protection appeared in the early state of ischemic stroke, and is associated with an improvement of the neurological deficits resulting from the arterial occlusion at PM and AM. 

Cerebral blood flow (CBF) derangements play key roles in the development of brain damage following cerebral ischemia [17]. Therefore, improved CBF has been proposed as one of the main strategies for limiting ischemic injury [18]. Intriguingly, when administration of galangin for treatment, there was a remarkable improvement in rCBF after MCAO, which was suspected to be associated with a inhibitor of the vascular Ca(v) 1.2 channels in the literature [13].

Recently, mitochondrial dysfunction has received great attention among the complex pathology of cerebral ischemia [19]. Mitochondria are involved in the ATP supply of cells, synthesis of key molecules and response to oxidative stress. Mitochondria contain many redox enzymes, which naturally occurring inefficiencies of oxidative phosphorylation and generate reactive oxygen species (ROS) in ischemic condition. Central nervous system (CNS) functions depend heavily on efficient mitochondrial function because the brain tissue has a high energy demand. The generation and presence of ROS and environmental factors may lead to encephalopathy, such as stroke [20]. EGb761 is an extract of the leaf of *Ginkgo biloba* L*.* that is neuroprotective against focal cerebral ischemic injury [21,22]. It has also been reported to protect against mitochondrial dysfunction [23] and inhibit the mitochondria-dependent caspase-apoptosis pathway [24]. Therefore, EGb761 was selected as the positive control for this study.

In the present study, MCAO induced marked mitochondrial dysfunction, including mitochondrial transition pore opening, membrane potential depolarization and ROS production [25,26,27]. Our data demonstrated that the increased ROS generation in mitochondria was markedly attenuated by galangin treatment. Moreover, galangin obviously favored the improvement of mitochondrial structure, as indicated by the attenuation of mitochondrial swelling and membrane viscosity, amelioration of the reduced mitochondrial membrane potential state, and inhibition of cerebral ischemia-induced mitochondrial dysfunction in a dose-dependent manner both at PM and AM.

Mitochondria play a key role in many apoptotic cascades and apoptotic cell death [28]. such as ial dysfuntion t AIF group at the dose of The mitochondrial pathway is closely related to cerebral ischemic injury. Following the mitochondrial protection, galangin sequentially inhibits caspase-dependent (cytochrome c and caspase-3) mitochondrial cell death pathway. In caspase-dependent manner, released cytochrome c activates the caspase cascade, causing cleavage of many proteins, DNA damage, and ultimately cell death [19]. It was reported that caspase inhibition abrogated cytochrome c release in ischemic brain injury [29]. Galangin at doses of 50 and 100 mg·kg^−1^ inhibited the activation of caspase-3 and cleavage of poly(ADP-ribose) polymerase (PARP), decreased Bax expression, increased Bcl-2 expression and maintained the balance of pro- and anti-apoptotic proteins when administrated 6 h after the MCAO. Moreover, the marked reduced release of cytochrome c from the mitochondria by galangin after the MCAO was also detected. 

## 3. Experimental

### 3.1. Animals

Adult male Sprague–Dawley rats weighing 250–270 g were obtained from the Animal Breeding Center of Beijing Vital River Laboratories Company (Beijing, China). All animals were housed individually at 22 ± 2 °C with a relative humidity of 50 ± 10% and a 12-h light/12-h dark cycle. The animals had free access to food and water. The experimental procedures were approved by the China Academy of Chinese Medical Science's Administrative Panel on Laboratory Animal Care. All animal experiments were performed in accordance with institutional guidelines and ethics.

### 3.2. Chemicals and Reagents

Galangin (purity 98.0%) was purchased from Nanjing Zelang Medical Technology Co., Ltd (Nanjing, China). The positive control, EGb761, was purchased from Dr. Willmar Schwabe (Karlsruhe, Germany). Resazurin sodium salt, 2,3,5-triphenyltetrazolium chloride (TTC), rhodamine 123, and 2',7'-dichlorofluorescin diacetate (DCFH-DA) were purchased from Sigma Chemical Co. (St. Louis, MO, USA). The rabbit polyclonal anti-Bax (P-19) and anti-active caspase-3 antibodies, the mouse monoclonal cytochrome c (A-8), the PARP-1 (F-2) and β-actin antibodies were purchased from Santa Cruz Biotechnology (Santa Cruz, CA, USA). Bcl-2 (50E3) was purchased from Cell Signaling. The JC-1 (lipophilic cation 5,5',6,6'-tetrachloro-1,1',3,3'-tetraethylbenzimidazolecarbocyanine iodide) Mitochondrial Membrane Potential Detection Kit was purchased from Beyotime Institute of Biotechnology (Haimen, China). The Na^+^-K^+^-ATPase and Ca^2+^-Mg^2+^-ATPase analysis kits were purchased from Nanjing Jiancheng Bioengineering Institute, China. The BCA Protein Assay Kit and the Super ECL Plus Western Blotting were purchased from Life Technologies (Carlsbad, CA, USA).

### 3.3. Animal Models and Experimental Protocol

After 48 h of acclimatization, the rats were anesthetized with chloral hydrate at a dose of 400 mg·kg^−1^ (i.p.). The rectal temperature was recorded and maintained at 37 ± 0.5 °C throughout the surgical procedure. The MCAO operation by the intraluminal filament method was performed according to a previous method with some modifications [30]. Briefly, 4-0 monofllament nylon suture with a round tip was inserted from the left external carotid artery into the lumen of the internal carotid artery to occlude the origin of the MCA. The rats were sacrificed 24 h after the MCAO. 

For the dose–response study, the rats were randomly divided into the following 6 groups (n = 10/group): sham group; vehicle control; positive control EGb761 group (4 mg·kg^−1^); and galangin-treated groups (25, 50 and 100 mg·kg^−1^). Galangin was dissolved in sterilize saline (containing 5% Tween 80) to make the stock solution. Dilutions were then prepared for the administration of the different doses. Galangin and the positive control, EGb761, were administered by intragastric administration (i.g.) 15 min prior to MCAO (PM) and 6 h after MCAO (AM). The sham and vehicle-treated rats were injected with physiological saline. Neurological defects were determined at 24 h after the MCAO followed by an examination of the brain infarct volume. The entire brain or the cortex was then removed and processed to detect cerebral infarct size and mitochondrial function. The regional cortical blood perfusion was also determined. 

### 3.4. Assessment of Neurological Defects

To reveal the effect of galangin on the neurological defects caused by the MCAO operation, the neurological defects were determined by a single researcher at 24 h after the MCAO. The researcher was blinded to the experimental treatment groups. The neurological behaviors were scored on 5-point scale as described previously [31]. 

### 3.5. Cerebral Infarct Size

The cerebral infract volumes measured with TTC staining were used for describing the severity of the cerebral ischemia. After 24 h of ischemia, the brains were quickly removed and sliced into 6 coronal sections 2-mm thick. The brain slices were treated with 2% TTC saline solution and incubated at 37.5 °C for 30 min, followed by 10% formalin fixation overnight according to a previously described method [32]. After staining with TTC, the normal tissue stained a rose red color, and the infarct tissue was white. The images of the stained slices were photographed and recorded. The adjusted infarct areas and both the hemisphere areas of each slice were determined by an image analysis system (Image-pro plus 6.0). The infarct volume of each slice was calculated as the infarct area × thickness (2 mm). The summation of the infarct volumes of all the brain slices was the total infarct volume. 

### 3.6. Regional Cortical Blood Perfusion

Laser speckle contrast imaging (LSCI) is a technique based on speckle contrast analysis that provides an index of blood flow [33,34,35]. A reduction in rCBF plays an essential role in ischemia-induced brain injury. To evaluate the effect of galangin, the rCBF was detected before and after the MCAO for each group. After deep anesthesia, each rat had a skin incision to expose the skull in a supine position prior to the test. The probe was positioned 10 cm above the detected frontoparietal cortex region of the brain left hemisphere. A round window of approximately 0.8 mm^2^ in size had been located left and down of the bregma, adjacent to the MCA area. The cerebral blood flow perfusion color image (Figure 3A.a and c), detected position image (Figure 3A.b), and blood flow time window (Figure 3A.d) were recorded.

Reduced rate of rCBF = (TOI1 − TOI2)/TOI1 × 100%

where the values of TOI1 and TOI2 were calculated as a percentage of the baseline value in the 15 min prior to and 30 min after the MCAO, respectively. Galangin and EGb761 were given intragastrically 15 min prior to MCAO.

### 3.7. Preparation of Rat Brain Mitochondria

The brain mitochondria were isolated from the left brain cortex tissue at 24 h after the MCAO. The fore-brain tissue was quickly removed and placed in ice-cold isolation buffer (250 mM sucrose containing 10 mM Tris-HCl, 0.5 mM Na_2_EDTA and 0.1% BSA, pH 7.1). The tissue was then washed to remove redundant blood and homogenized [20% (w/v)]. The nuclei and cell debris were sedimented by centrifugation at 600 rpm for 3 min and 1000 rpm for 5 min and then discarded. The supernatant was subjected to further centrifugation at 10,000 rpm for 8 min. The mitochondrial pellet was washed by gently resuspending the pellet in isolation medium and then centrifuging at 10,000 rpm for 8 min [36]. Finally, the mitochondria were resuspended in the above buffer to give a concentration of 10 mg·mL^−1^. All procedures were performed at 4 °C. The mitochondrial protein concentration was determined by BCA assay (Life Technologies). 

### 3.8. Measurement of Mitochondrial Viability

Resazurin is a sensitive indicator of mitochondrial function, which is hydrolyzed to fluorescent resorufin by mitochondrial activity related enzyme [37]. Mitochondria (50 μg proteins) were added into a 96-well plate and incubated with 5 μM resazurin at 37 °C. The fluorescence intensity was measured with a microplate reader (SpectraMax M5, Molecular Devices, Sunnyvale, CA, USA) set to a 530 nm excitation wavelength and 590 nm emission wavelength after one hour incubation. Samples containing equal amounts of mitochondrial protein that had been heated to 100 °C for 10 min prior to the addition of resazurin were used to obtain the background signal. The stronger the fluorescence intensity of resorufin, the better the mitochondrial viability it results.

### 3.9. Measurement of Mitochondrial Swelling

Mitochondrial swelling following a PT pore opening assayed by measuring the decrease in absorbance at 520 nm at 25 °C according to the method of Tian [38]. The turbidity of the reaction mixture reflected the degree of mitochondrial swelling. The assay mixture contained freshly prepared mitochondrial protein (50 μg protein), 70 mM sucrose, 10 mM succinate, 5 mM Hepes, 1 mM Na_2_HPO_4_, 210 nM mannitol, 2.7 μM rotenone, and 1 μg·ml^−1^oligomycin A (pH 7.4). A kinetic decrease in the absorbance was recorded over a period of 10 min in 200 μL medium using the microplate reader described above. The control group had the same amount of mitochondria without rotenone and oligomycin A. The detected absorbance had a good linear relationship and the absolute slope was used to compare each group [39]. The greater the slope, the greater the mitochondrial swelling it gets.

### 3.10. Measurement of Mitochondrial Membrane fluidity

Membrane fluidity was measured by the fluorescence polarization (FP) method with a probe, diphenylhexatriene (DPH), as previously described by Hirano [40]. DPH (5 µM) was added to freshly prepared mitochondria in medium (250 mM sucrose containing 10 mM Tris-HCl, 0.5 mM Na_2_EDTA and 0.1% BSA, pH 7.1), which were incubated at 37 °C for 30 min to allow probe incorporation. The mp value was monitored at 37 °C with the microplate reader described above. The excitation wavelength was 362 nm, and the emission wavelength was 432 nm. To identify the effect of galangin, η, which is the representation of the coefficient of viscosity of the mitochondrial membrane, was calculated according to the formula:

η = 2P/(0.46 − P)

where the values of P were calculated according to the formula P = 1000 × mP. The higher the value of η, the lower the fluidity of the mitochondrial membrane it shows.

### 3.11. Measurement of Mitochondrial Transmembrane Potential (Δψm)

#### 3.11.1. Rhodamine 123 Method

Changes in brain mitochondrial Δψm were measured in the presence of rhodamine 123 (Rh123)as described previously [41]. The excitation and emission wavelengths for Rh123 were 503 and 527 nm, respectively, using a SpectraMax M5 Microplate Reader. Δψ_m _was assessed based on the quantitation of Rh123 quenching. A low Δψ_m_ level corresponds to a higher value of Rh123 fluorescence. The basal fluorescence ([Rh123] total) was determined before adding the mitochondria. The mitochondria (0.5 mg protein) were then added and incubated in 150 μL buffer (150 mM sucrose, 5 mM MgCl_2_·6H_2_O, 5 mM succinate, 5 mM KH_2_PO_4_, 20 mM Hepes, 2.7 μM rotenone, and 0.5 μM rhodamine 123, PH 7.4) for 1 h. following the uptake of Rh123, the fluorescence quenching of Rh123 was measured ([Rh123] out). The mitochondrial membrane potential was calculated with the Nernst equation [42].

#### 3.11.2. JC-1 Method

As another method, the JC-1 Mitochondrial Membrane Kit was also used to monitor the changes in ΔΨm. JC-1 is an ideal fluorescent probe for the detection of ΔΨm. When the ΔΨm is high, JC-1 aggregates in the matrix of the mitochondria, which produces red fluorescence. By contrast, when the ΔΨm is low, JC-1 exists in monomeric form and produces green fluorescence. The ratio of the red/green fluorescence intensity at 590 nm to that at 530 nm was used to measure the depolarization of the mitochondrial membrane [43].

### 3.12. Measurement of Reactive Oxygen Species (ROS) Production in Mitochondria

Reactive oxygen species (ROS) production in rat brain mitochondria was monitored by the fluorescent probe DCFH-DA [42] promptly after mitochondria were prepared. Intracellular ROS can oxidize DCFH-DA to dichlorofluorescein (DCF), an intensely fluorescent chemical. Mitochondria isolated from different groups (0.5 mg protein) were incubated with 10 μM DCFA at 37 °C for 60 min, and the fluorescence intensity of DCF was measured at an excitation wavelength of 488 nm and emission wavelength of 527 nm in the microplate reader.

### 3.13. Western Blot of Apoptosis Related Proteins

#### 3.13.1. Cortex Proteins

The rat brain homogenate in ice-cold lysis buffer containing 150 mM NaCl, 25 mM Tris–HCl, 1 mM EGTA, 1 mM EDTA, 1% Triton X-100, 0.5% NP-40, 1 μg·mL^−1^ aprotinin, 1 μg·mL^−1^ leupeptin, and 1 mM PMSF, pH 7.4, was used for the Western blot experiments [20% (w/v)]. The homogenate was incubated on ice for 30 min and then centrifuged at 12,000 rpm for 20 min at 4 °C. The supernatant was collected, and the protein concentrations of the extracts were measured by BCA assay. The protein samples in the supernatant were resolved by SDS-polyacrylamide gel electrophoresis (SDS-PAGE) and electrotransferred to PVDF membranes [42]. The membrane was incubated with the respective primary antibodies against Bcl-2 (1:1,000), Bax (1:1,000), activated caspase-3 (1:1,000), or PARP (1:1,000) overnight at 4 °C. The antibody for β-actin (1:5,000) served as the loading control. Finally, the membrane was incubated with horseradish peroxidase-conjugated secondary antibody. The protein bands were visualized using the ECL Western blotting detection kit (Life Technologies). The relative intensities of the bands were quantified by densitometric analysis. The densitometric plots of the results were normalized to the intensity of the actin band.

#### 3.13.2. Mitochondrial Proteins

The release of mitochondrial cytochrome c was determined by Western blot experiments according to the method of He [42]. The rat brain homogenate in ice-cold lysis buffer mentioned above [20% (w/v)] was centrifuged at 1000 rpm for 10 min, and the resulting supernatant was centrifuged at 10,000 rpm for 10 min. The pellet was the mitochondrial fraction. The supernatant was then re-centrifuged at 10,000 rpm for 1 h at 4 °C. The resulting supernatant was used as the cytosolic fraction. The forty microgram proteins in pellet and supernatant were prepared and immunoblotted with cytochrome c antibody (1:1,000). The relative intensities of the bands were also quantified by densitometric analysis. The densitometric plots of the results were normalized to the intensity of the actin band. 

### 3.14. Statistical Analyses

The data were expressed as the means ± SD. The statistical significance of differences between groups was determined by one-way analysis of variance (ANOVA). *p* value < 0.05 was considered statistically significant.

## 4. Conclusions

In conclusion, this study provided comprehensive evidence supporting the potential therapeutic effect of galangin in treatment of acute ischemic stroke in a dose-dependent manner both at PM and AM. Once the rCBF was reduced, the mitochondrial dysfunction and caspase-dependent mitochondrial cell death pathway occured. Although the approaches where galangin works may not be limited to one pathway, yet the results supported the idea that galangin played a direct protection against ischemic injury, and the therapeutic effect could be expressed via mitochondrial protection and inhibition of the caspase-dependent mitochondrial cell death pathway. Furthermore, the protective effects of galangin might also be due to the improvement in rCBF after MCAO. The animal experimental results showed that galangin was a promising neuroprotective agent after acute ischemic stroke and more study is needed for clinical use in the future. 
